# Atypical antipsychotics induce human osteoblasts apoptosis via Wnt/β-catenin signaling

**DOI:** 10.1186/s40360-019-0287-9

**Published:** 2019-02-12

**Authors:** Peifan Li, Yiming Wang, Xingde Liu, Zhen Zhou, Jun Wang, Haiyan Zhou, Lei Zheng, Lixia Yang

**Affiliations:** 1 Department of Psychiatry, Hospital Affiliated to Guizhou Medical University, Guiyang, 550004 Guizhou China; 2 Neuroelectrophysiological testing center, Hospital Affiliated to Guizhou Medical University, Guiyang, 550004 Guizhou China; 3 Undergraduate mental health education and counseling center, Guizhou Medical University, Guiyang, 550004 Guizhou China; 4 Department of Cardiology, Hospital Affiliated to Guizhou Medical University, Guiyang, 550004 Guizhou China; 5 Clinical research center, Hospital Affiliated to Guizhou Medical University, Guiyang, 550004 Guizhou China

**Keywords:** Atypical antipsychotics, Osteoblasts, β-catenin, Bcl-2, Mcl-1, Bax, Cleaved-Caspase3

## Abstract

**Background:**

There is evidence that atypical antipsychotics (APs) increase risk of osteoporosis in schizophrenia patients, however the mechanism is unclear. The aim of the study was to explore the molecular mechanisms about Wnt/β-catenin signal pathway underlying the osteal side effects of APs.

**Methods:**

We cultured human osteoblast cell line hFob1. 19 (OB) treatments with olanzapine, risperidone, amisulpride, aripiprazole or resveratrol in vitro. OB cells viability was detected by cell viability assay. OB cells apoptosis was analyzed by flow cytometry (FCM). Further apoptosis-related marker and β-catenin expression was analyzed by Western blot and Immunofluorescence analysis.

**Results:**

Compared with the control group, proliferation of OB cells decreased and apoptosis rates of OB cells increased significantly in APs group (*p* < 0.05). There were a reduced level of Bcl-2, Mcl-1 (antiapoptotic marker) and an elevated level of Bax, Cleaved-Caspase3 (proapoptotic marker) in APs group (*p* < 0.05). Simultaneously, β-catenin expression decreased in cytoplasm and nucleus (*p* < 0.05). Compared with the just APs group, the apoptosis rates decreased and β-catenin expression increased significantly in resevratrol combined with APs group (*p* < 0.05). Correlation analysis showed positive correlation between β-catenin expression and the apoptotic rate in OB cells (r = − 0.515, *p* < 0.05).

**Conclusions:**

APs cause OB cells apoptosis relating to Wnt/β-catenin signaling while resevratrol could reverse this phenomenon. Our study could lay the foundation for overcoming the APs-induced osteal side effects to improve the life quality of schizophrenia patients.

## Background

Schizophrenia has a prevalence of ~ 1% worldwide and brings these individuals suffering from chronic symptoms and disabilities, while antipsychotic drugs maintain the standard for schizophrenia treatment [1, 2]. With the wide use of antipsychotic drugs, especially atypical antipsychotics (APs), the focus of treatment on schizophrenia gradually shifted from controlling symptoms to improving the quality of patients life [3, 4]. APs could be more effective in treating positive symptoms (e.g., hallucinations and delusions), negative symptoms (e.g., impaired motivation and reduction in spontaneous speech), cognitive symptoms but fewer movement disorders. Unfortunately, there is evidence that long-term treatment with APs would carry a higher risk of clinically significant metabolic adverse events, including weight gain, metabolic syndrome, lipid and glycemic values fluctuation, diabetes mellitus and insulin resistance [5–9]. Furthermore, this clinical observation supported schizophrenia patients with long-term exposure to APs had decreased bone mineral density (BMD) [10]. Mortality in schizophrenic patients with major fractures increased by 54% compared with the schizophrenic patients without fractures [11], and the hip fractures in schizophrenia patients would cause mental state and ambulatory worsening [12]. It is a pity that the basic molecular mechanism by which antipsychotic drug treatment leads to a negative effect on bone is unclear.

Low BMD and microarchitectural alterations of bone tissue be caused by imbalance between osteoblasts rebuilt and resorbed bone [13]. Osteoblastic bone formation is one of the important factors in bone mass maintenance [14]. From the level of molecular biology, a number of signal pathways include BMP-Smads, Wnt/β-catenin, Notch, Hedgehog, FGF et al. are involved in the osteoblast regulation [15]. Wnt/β-catenin signaling regulated osteoblast apoptosis is a new insight. These study reported that β-catenin accumulated in the cytoplasm and subsequently translocates into the nucleus [16], where it associated with the T cell factor/ lymphocyte growth factor (TCF/LEF) into the complex, thus regulated the apoptotic gene transcription [17]. Wnt/β-catenin signaling pathway may be a target for avoiding the APs-induced osteal side effects. However, the classic activator of β-catenin, Lithium chloride (LiCl), is a toxic compound which is harmful to humans [18]. Fortunately, resveratrol, a natural polyphenolic compound and abundantly found in plant foods, is also thought to be involved in Wnt/β-catenin signaling pathway activation [19, 20]. If resveratrol is effective, it will be more suitable for preventing osteoporosis associated with Aps.

Hence, the aim of this study was to measure the viability, apoptosis rate, apoptosis-related protein and β-catenin in OB cells to determine whether APs-induced apoptosis in OB cells as well as that involved in Wnt/β-catenin signal pathway and whether resevratrol reduce the apoptosis through Wnt/β-catenin signal pathway. Our study could provide evidence about antipsychotics have adverse effects on osteoblasts. Additionally, it could lay the foundation for overcoming the APs-induced osteal side effects.

## Methods

### Chemicals and antibodies

The following four antipsychotics drugs Olanzapine, Risperidone, Amisulpride and Aripiprazole were obtained from Sigma-aldrich (Sigma, USA). Fetal bovine serμM (Gibco BRL); RPMi-1640 medium (Gibco BRL); Dimethyl Sulfoxide (DMSO; Sigma-Aldrich, St. Louis, MO, SUA); Cell counting kit-8 (CCK8) (Tongren, Tokyo, Japan) Annexin V-Fuorescein Isothiocyanate (FITC)/Propidium Iodide (PI) apoptosis detection kit (BD Biosciences, San Jose, CA, USA); Primary antibodies such as β-actin, BAX, BCL-2, MCL-1,Caspase-3, Cleaved-Caspase3 and β-catenin were obtained from Cell Signaling Technology (Beverly, MA, USA); Secondary antibodies for western blot analysis were obtained from Santa Cruz Biotechnology (Inc, CA, USA) and Cell Signaling Technology (Beverly, MA, USA).

### Cell lines and cell culture conditions

The human osteoblast cell line hFob1. 19 (OB) was purchased from ATCC. The cell line was cultured at 37 °C in a 5%CO_2_ saturated humidity in RPMi-1640 medium supplemented with 20% fetal bovine serum (Gibco BRL; Life Technologies, Carlsbad, CA, USA), penicillin (100 U/ml), and streptomycin (100 μg/ml).

### Cell viability assay

Different groups of OB cells were seeded at the density which 5000 cells per well in 96-well plates. There were 3 duplicated wells, and negative control wells and blank wells were set. After overnight incubation, the cells were treated with Olanzapine (20, 40, 60, 80, 100 and 120 μM), Risperidone (20, 40, 60, 80, 100 and 120 μM), Amisulpride (20, 40, 60, 80, 100 and 120 μM), Aripiprazole (2.5, 5, 15, 10, 20 and 40 μM) or 0.1%DMSO. After 12, 24 or 48 h, added CCK-8 10ul per well. Then incubation for 3 h, the absorbance of each well was measured at the wavelength of 490 nm by ELIASA.

### Apoptosis analysis

OB cells treated with Olanzapine, Risperidone, Amisulpride, Aripiprazole, resveratrol or 0.1%DMSO for 24 h. The cells were harvested and washed with phosphate-buffered saline (PBS), and then cell pellets were harvested and stained with an Annexin V-FITC/PI apoptosis kit (BD Biosciences, San Jose, CA, USA) according to the manufacturer’s instruction. After being stained at room temperature for 15 min in the dark, the cells were measured with FCM and the Cell Quest software (BD Biosciences). The cells were measured with FCM and the Cell Quest software (BD Biosciences).

### Protein extraction

Total protein extraction: OB cells were digested with trypsin and centrifuged, and then the supernatant was removed and washed by PBS. Joined protein cracking liquid RAPI and centrifuged at 4 °C. The supernatant is the total protein. Extraction of cytoplasm protein and nuclear protein: cytoplasm protein extraction reagent and nuclear protein extraction reagent respectively were added to PMSF, made final concentration of PMSF was 1 mM. OB cells were digested with trypsin and centrifuged, and then the supernatant was removed and washed by PBS. Added 200 μL mixture of cytoplasm protein extraction reagents and PMSF per 20 μL precipitate, high-speed vortex made precipitation fully spread out into suspense and centrifugation at 4 °C, the supernatant is cytoplasmic protein. Added 50–100 μL mixture of nuclear protein extraction reagent and PMSF in the precipitate, high-speed vortex made precipitation fully spread out into suspense and centrifugation at 4 °C, the supernatant is nuclear protein.

### Western blot analysis

Western blot analysis (WB) was performed to analyze protein expression and activation after cells were treated with Olanzapine (40 μM), Risperidone (40 μM), Amisulpride (30 μM) and Aripiprazole (10 μM). Briefly, cells were washed in PBS, collected and then lysed in RIPA buffer (radio immunoprecipitation assay buffer, 50 mM Tris-HCl; 150 mM NaCl; 0.1% SDS; 0.5% Na-deoxycholate; 1% NP40) containing proteinase inhibitor cocktail and phosphatase inhibitor cocktail (Roche Applied Science, Indianapolis, IN, USA). The lysate was centrifuged at 12000 rpm at 4 °C for 15 min, and equal protein lysate was used for Western blot analyses. For WB, 50 μg of total cell lysate was subjected to SDS-PAGE. The proteins were then transferred to a polyvinylidene difluoride membrane (Pall Corp, Ann Arbor, MI), and the membranes were blocked in 1 × PBS, 0.1% Tween-20 and 5% skim milk. After blocking, the membranes were incubated with primary antibodies diluted 1:1000 in 1 × PBS, 5% skim milk and 0.1% Tween-20 overnight at 4 °C. Immunodetection was performed by the Western blotting Luminol Reagent (Santa Cruz Biotechnology Inc. CA, USA). Actin immunoblotting was performed to verify that equal protein had been loaded in each lane. Their optical density was analyzed with Quantity One software. The expressions of target proteins were normalized to β-actin.

### Immunofluorescence analysis

OB cells were plated confocal microscopy special cell culture dish, and incubated with Olanzapine (40 μM), Risperidone (40 μM), Amisulpride (30 μM) and Aripiprazole (10 μM) or 0.1%DMSO for 24 h. Then the cells were rinsed in PBS and fixed by incubation with 4% formaldehyde for 20 min at room temperature. After washing with PBS, cells were permeabilized with PBS containing 0.25% Triton X-100. The cells were blocked in PBS containing 5% BSA for 30 min. Next, with further washing cells were incubated with a rabbit anti-β-catenin monoclonal antibody, overnight at 4 °C temperature. After washing with PBS, cells were incubated with FITC-conjugated goat anti-rabbit antibody (1:200) for 30 min. Then, the nucleus was stained with DAPI (4, 6-diamidino-2-phenylindole) and cells were observed under a 20× field of view by confocal microscopy (LSM710, ZEISS).

### Statistical analysis

Each experiment or assay was performed at least three times, and representative examples were shown. Data are reported as means ± SEM. Statistically significant differences between the treated groups were calculated using Student’s t-test. Differences were considered statistically significant at *P* < 0.05. Pearson correlation was used to assess relationship.

## Results

### Inhibition of OB cells viability after treatment with APs

**Fig. 1:**
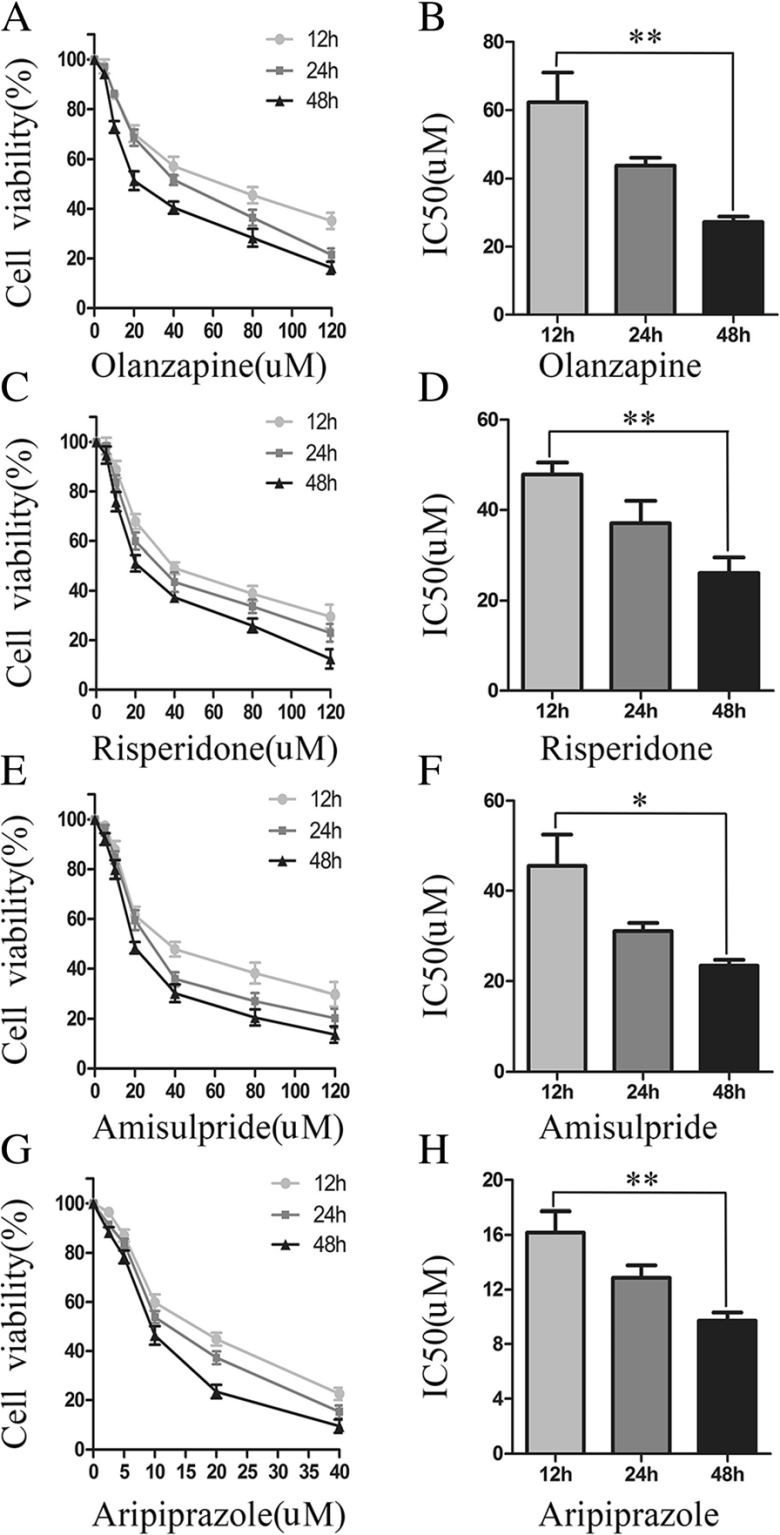
Inhibition of OB cells viability after APs treatment. **a**, OB cell viability was examined by CCK-8 assay after exposured to olanzapine (0.1%DMSO, 10, 20, 40, 80 or 120 μM), risperidone (0.1%DMSO, 10, 20, 40, 80 or 120μM), amisulpride (0.1%DMSO, 10, 20, 40, 80 or 120μM) or aripiprazole (0.1%DMSO, 2.5, 5, 10, 20 or 40 μM) at 12h, 24h or 48h (**a**, **c**, **e**, **g**). Bar graph indicated the IC-50 values of olanzapine, risperidone, amisulpride, aripiprazole in 1640 mediuM at 12, 24 and 48 hours (**b**, **d**, **f**, **h**). The data were calculated with GraphPad Prism. *P < 0.05; **P < 0.01

To determine the effect of APs on proliferation of OB cells, we examined cell viability using CCK8 assay after the four APs (olanzapine, risperidone, amisulpride and aripiprazole) treatment respectively. Our results showed that the proliferation of OB cells decreased significantly compared with the control group (Fig. 1 a, c, e, g). In Bar graph 1 B, the IC50 values of olanzapine were 62.29 ± 12.23 μM, 43.73 ± 3.27 μM, and 27.24 ± 2.35 μM at 12 h, 24 h and 48 h. In Bar graph 1 D, the IC50 values of risperidone were 47.82 ± 3.78 μM, 37.06 ± 7.01 μM, and 26.13 ± 4.82 μM at 12 h, 24 h and 48 h. In Bar graph 1 F, the IC50 values of amisulpride were 45.51 ± 9.96 μM, 31.10 ± 2.53 μM, and 23.04 ± 1.90 μM at 12 h, 24 h and 48 h. In Bar graph 1 H, the IC50 values of aripiprazole were 16.18 ± 2.19 μM, 12.86 ± 1.30 μM, and 9.73 ± 0.82 μM at 12 h, 24 h and 48 h. Our data indicated that APs had an inhibitory effect on OB cells in a dose-dependent and time-dependent manner.

### APs-induced apoptosis in OB cells

**Fig. 2:**
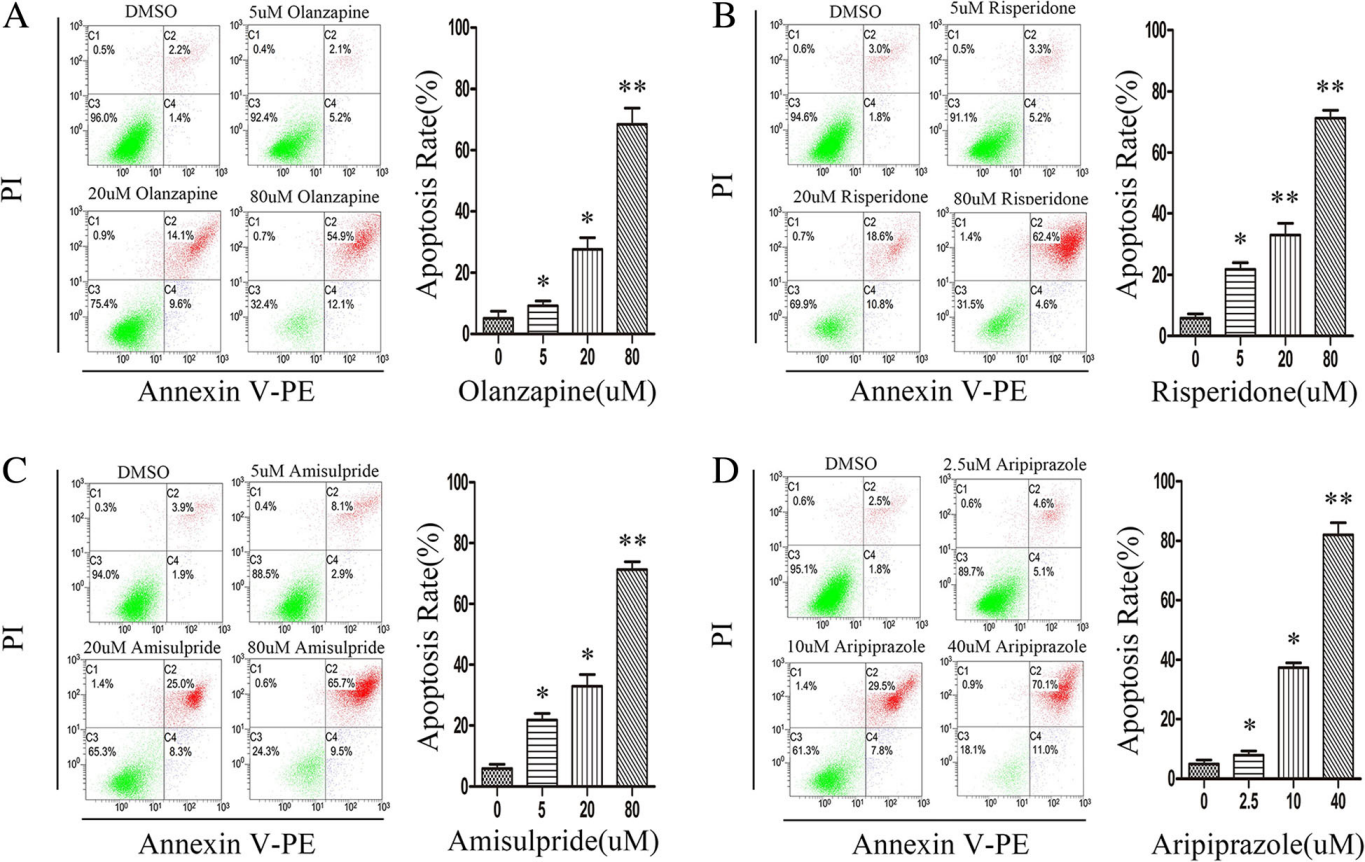
Effect of APs on OB cells apoptosis. OB cells treated with olanzapine (0.1%DMSO or 5, 20, 80, μM), risperidone (0.1%DMSO or 5, 20, 80, μM), amisulpride (0.1%DMSO or 5, 20, 80, μM) or aripiprazole (0.1%DMSO or 2.5, 10, 40, μM) incubated in 1640 medium at 24 h and the apoptosis of OB cells were analyzed by flow cytometry (**a-d**). Bar graph indicates the percent of Annexin V-positive cells (apoptotic cells) of experiments three times. The data were calculated with GraphPad Prism. *P < 0.05; **P < 0.01

To further determine whether the four APs induce apoptosis in OB cells, we analyzed OB cells apoptosis by flow cytometry. The apoptosis rates of OB cells treated with olanzapine were 3.83 ± 2.34% (0.1% DMSO), 16.05 ± 2.12% (5 μM), 25.63 ± 3.90% (20 μM), 71.43 ± 5.23% (80 μM) (Fig. 2 a). The apoptosis rates of OB cells treated with risperidone were 4.73 ± 0.90% (0.1% DMSO), 17.67 ± 4.15% (5 μM), 29.37 ± 1.25% (20 μM), 66.70 ± 4.26% (80 μM) (Fig. 2 b). The apoptosis rates of OB cells treated with amisulpride were 5.80 ± 2.40% (0.1% DMSO), 21.83 ± 3.68% (5 μM), 32.93 ± 6.65% (20 μM), 71.26 ± 4.47% (80 μM) (Fig. 2 c). The apoptosis rates of OB cells treated with aripiprazole were 4.93 ± 2.31% (0.1% DMSO), 7.87 ± 2.44% (2.5 μM), 37.37 ± 3.78% (10 μM), 82.07 ± 7.10% (40 μM) (Fig. 2 d). Compared with the control group, apoptosis rates of OB cells treated by APs were significantly increased in a dose-dependent manner.

### The broken balance between proapoptotic and antiapoptotic markers causing apoptosis

**Fig. 3:**
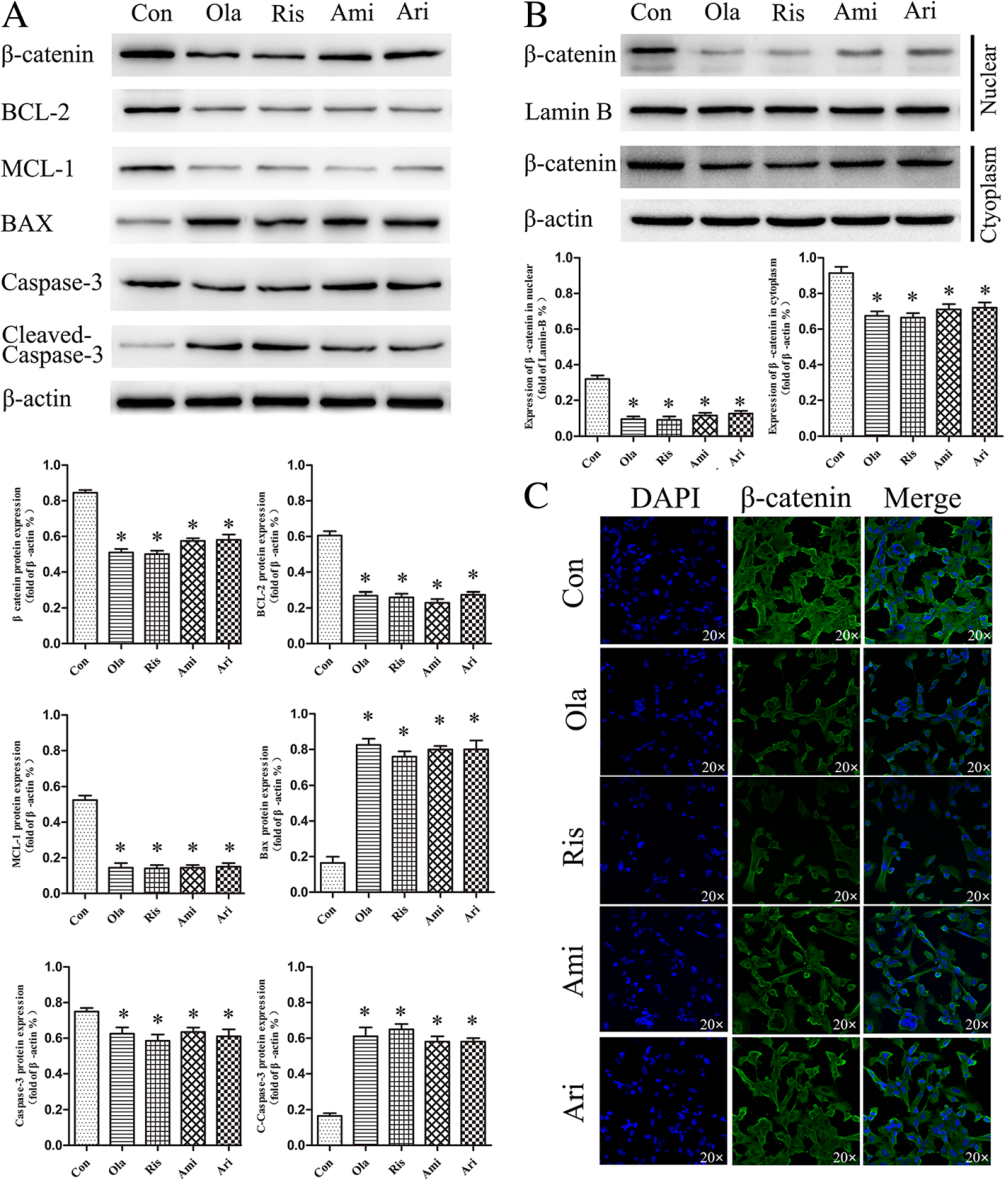
APs-induced apoptosis related to inhibition of Wnt/β-catenin signaling in OB cells. **a**, apoptosis-related protein and β-catenin protein expression was measured by western blot after exposured to olanzapine (Ola, 40 μM), risperidone (Ris, 40 μM), amisulpride (Ami, 30 μM) or aripiprazole (Ari, 12 μM) treatment at 24 h in OB cells. **b**, Nuclear and cytoplasmic protein of β-catenin were analyzed by western blot after exposured to olanzapine (40 μM), risperidone (40 μM), amisulpride (30 μM) or aripiprazole (12 μM) at 24 h in OB cells. **c**, The different expression of β-catenin protein between nuclear and cytoplasm was determined by Immunoflourescence analysis after exposured to olanzapine (40 μM), risperidone (40 μM), amisulpride (30 μM) or aripiprazole (12 μM) at 24 h in OB cells. The data were calculated with GraphPad Prism. *P < 0.05; **P < 0.01

We had previously shown that treatment with the APs induced apoptosis rates upregulation in OB cells. To gain insight into the mechanism of APs-induced apoptosis in OB cells, We measured apoptotic protein Bcl-2, Mcl-1, Bax which belong to B cell lymphoma 2 (BCL­2) family by WB. We found a reduced level of Bcl-2, Mcl-1 (antiapoptotic protein) and an elevated level of Bax (proapoptotic protein) after olanzapine (40 μM), risperidone, amisulpride and aripiprazole treatment compared with the control group. Additionally, Cleaved Caspase-3 increased while Caspase-3 decreased compared with the control group (Fig. 3 a). In the four treatment groups, olanzapine and risperidone had the stronger inhibitory effect on β-catenin than amisulpride and aripiprazole at the IC50 concentration (Fig. 3 a).

### The correlation between β-catenin and apoptotic markers

The importance of inhibition Wnt/β-catenin signaling had been confirmed in osteopenia related to osteoblast [16]. Our study previously indicated that APs increased apoptosis rate of OB cells. Together, we hypothesized that APs drugs might cause osteoblast apoptosis through Wnt/β-catenin signaling. To test this possibility, we measured protein expression of β-catenin after APs treatment respectively by WB. We found that β-catenin protein expression decreased compared with control group (Fig. 3 a). Since the functions of β-catenin depended on its expression in nucleus [17], subcellular fractionation immunoflourescence analysis and WB were performed. β-catenin was showed respectively decreased in cytoplasm and nuclear of OB cells after the four APs treatment (Fig. 3 c and b). These results suggested that inhibition of Wnt/β-catenin signaling was connected with increased apoptosis.

### Heightened Wnt/β-catenin signaling prevented APs-induced apoptosis

**Fig. 4:**
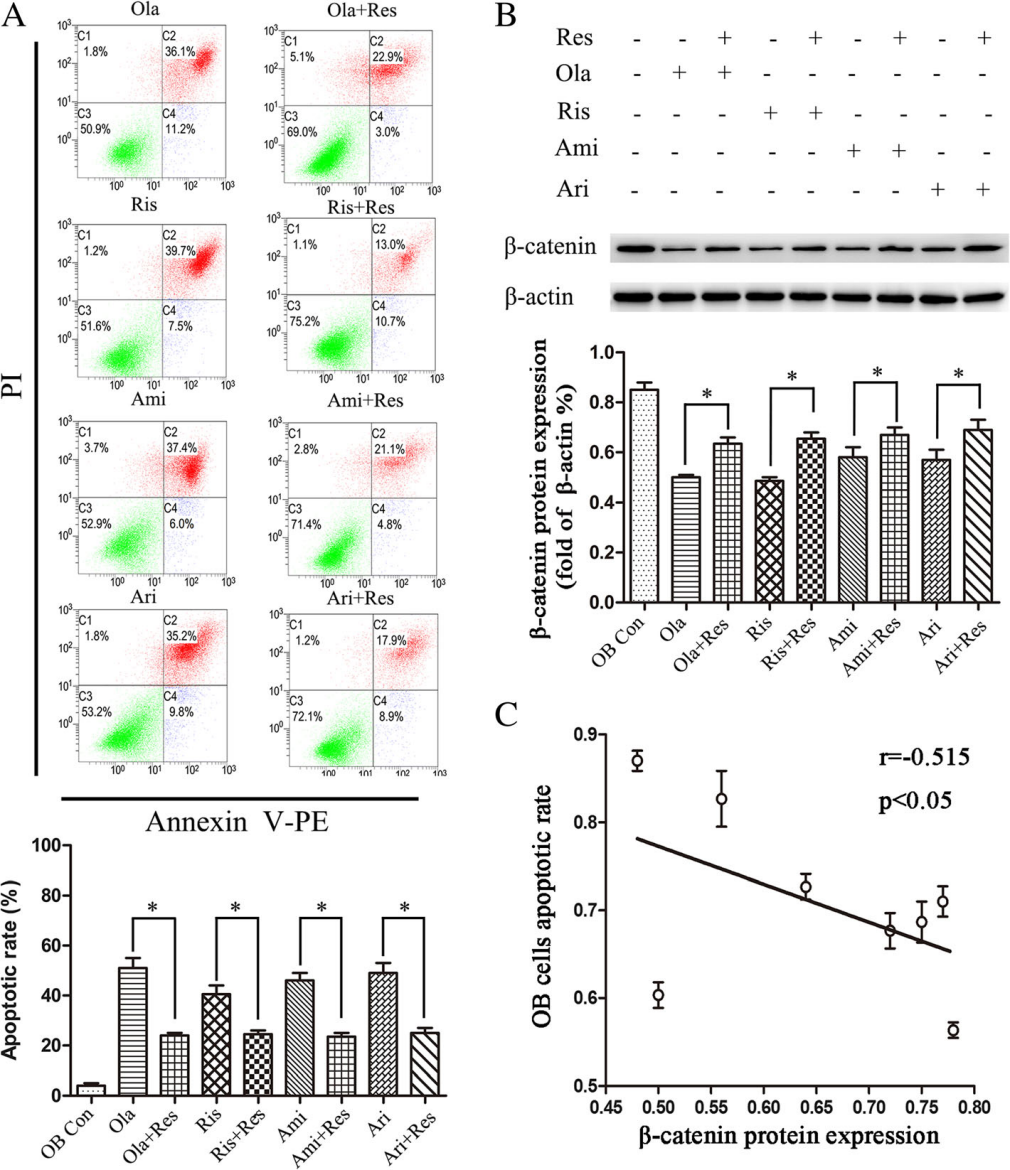
Activation of Wnt/β-catenin signaling to protect osteoblasts. **a**, the apoptosis of OB cells were analyzed by flow cytometry after olanzapine (40 μM), risperidone (40 μM), amisulpride (30 μM), aripiprazole (12 μM) with or without resveratrol (Res, 50 μM) treatment at 24 h. Bar graph indicates the percent of Annexin V-positive cells (apoptotic cells). **b**, β-catenin protein expression was measured by WB after olanzapine (40 μM), risperidone (40 μM), amisulpride (30 μM), aripiprazole (12 μM) with or without resveratrol (50 μM) treatment at 24 h in OB cells. The data were calculated with GraphPad Prism. **c**, Correlation between OB cells apoptosis rate and β-catenin protein expression in OB cells. *P < 0.05; **P < 0.01

Based on the ability of resveratrol to activate the β-catenin/TCF-mediated transcriptional activity [21], we selected it as activator of β-catenin. To study the effects of Wnt/β-catenin signaling on apoptosis rate of OB cells, we examined the apoptosis rate of OB cells again after resevratrol combined with APs treatment or just APs treatment. The apoptosis rate of olanzapine group was 51.2 ± 2.3%. The apoptosis rate of olanzapine combined with resevratrol group was 22.1 ± 0.3%. The apoptosis rate of risperidone group was 45.6 ± 2.5%. The apoptosis rate of risperidone combined with resevratrol group was 22.8 ± 0.5%. The apoptosis rate of amisulpride group was 47.3 ± 2.7%. The apoptosis rate of amisulpride combined with resevratrol group was 21.7 ± 0.6%. The apoptosis rate of aripiprazole group was 52.7 ± 2.5%. The apoptosis rate of aripiprazole combined with resevratrol group was 22.8 ± 0.8%. Compared with the just APs group, the apoptotic rates of APs combined with resevratrol group decreased significantly (Fig. 4 a). In the WB, β-catenin expression in APs combined with resevratrol group was significantly higher than just APs group (Fig. 4b). Correlation analysis showed negative correlation between β-catenin expression and the apoptotic rate in OB cells (Fig. 4c) (*r* = − 0.515, *p* < 0.05).

## Discussion

This study demonstrated that APs induced OB cell apoptosis. Further, the study provided evidence that expression of apoptosis-related proteins was adjusted after APs treatment. However, the current study suggest that APs may induce low BMD by sustaining elevate the secretion of prolactin and subsequently inhibit estrogen secretion as their dopamine D2 receptor-blocking effect [22]. High prolactin level being negatively correlation with estrogen concentration strongly relates to BMD loss [23]. A clinical trial showed that the prolactin-raising medication group (*n* = 26) had higher rate of bone pathology compared with the control group (*n* = 12). Unfortunately, the detailed mechanisms have not been investigated, particularly direct impact of APs on osteoblast. Just a few experiments superficially described one of APs impaired viability and function of osteoblasts [24–26]. Our study is the first to suggest a novel mechanism of APs induced apoptosis in OB cells.

The study identified the reduced level of Bcl-2, Mcl-1 and elevated level of Bax by WB. It is well known that BCL­2 gene family is fundamental to the balance between cell survival and death, which encodes multiple proapoptotic and antiapoptotic proteins to regulate the intrinsic apoptosis pathway [27, 28]. Increased proapoptotic Bax bound to the mitochondrial outer results in membrane outer membrane permeabilization (MOMP) and the release of cytochrome c [29]. Trend in the apoptosis inducing signal, leaved caspase-3, a key executive in the process of apoptosis [24], hydrolyzes the target substance within the cell to degrade intracellular protein, resulting in irreversible death [30]. While the decreased expression of antiapoptotic protein Bcl-2, Mcl-1 reduced the effect which blocked the proapoptotic protein-mediated MOMP lead to increased apoptosis [31]. In Our work, cleaved caspase-3 expression increased also together with the current study.

In addition, our work highlighted the connection between Wnt/β-catenin signaling and atypical antipsychotic-induced apoptosis. The data indicated that Wnt/β-catenin signaling suppression is related to proapoptotic Bax, Caspase-3 up-regulation and antiapoptotic Bcl-2, Mcl-1 down-regulation. The Wnt/β-catenin signaling is an important modulator in bone homeostasis because it robustly promoted mesenchymal stem cells differentiated into osteoblasts and osteogenesis [32]. Previous studies also showed that wnt/β-catenin signaling involved in protecting osteoblasts and osteocytes from apoptosis [33–35]. In parallel with our study, a cell culture experiments showed that activating β-catenin by LiCl or Wnt1 inhibited the H2O2-mediated cell viability decreased and restored the mitochondrial Bcl-2/Bax ratio in human osteoblasts [36].

Interestingly, our study indicated that resevratrol could reverse the drop of β-catenin even reduced APs induced-apoptosis. Resevratrol had protective effect on inflammatory, oxida, aging, carcinoma had been reported [37–39]. Indeed, resevratrol was shown promote osteoblast proliferation and differentiation from multipotent mesenchymal cells [40]. Recent studies also demonstrated that resveratrol promoted osteoblast proliferation via activation of Wnt/β-catenin signaling [41]. More recently, the published data indicated that resevratrol upregulated sirt1 in osteoblast and volved in the control of osteoblast proliferation, differentiation and apoptosis [42]. Of note, sirt1 as a NAD-dependent histone deacetylase be provided controlling anxiety in mice [43]. There was also a link between the expression of sirt1 gene and depression [44]. Herein, we venturesome hypothesized whether resevratrol could be combined with APs to resist APs-induced low BMD and to relieve emotional disorders with schizophrenia patients. We would test this interesting hypothesis in the future experiments.

Taken together, our study suggested that APs may induce osteoblast apoptosis related to Wnt/β-catenin signal pathway inhibition while resevratrol could reverse this phenomenon. In addition to APs, common risk factors for osteoporosis including smoking, physical inactivity, polydipsia, alcohol misuse, hypogonadism, a family or personal history of fractures, and vitamin D deficiency are all increased in schizophrenia patients [45]. Studying the side effects of atypical antipsychotics, it is not to deny APs but to use it rationally in clinical such as schizophrenia patients with high osteoporotic risk need special attention with APs treatment. What is more, rational use of APs and active management of BMD loss in schizophrenia patients who have antipsychotic-associated bone disease could delay or even reverse this process [46].

## Conclusions

**Fig. 5:**
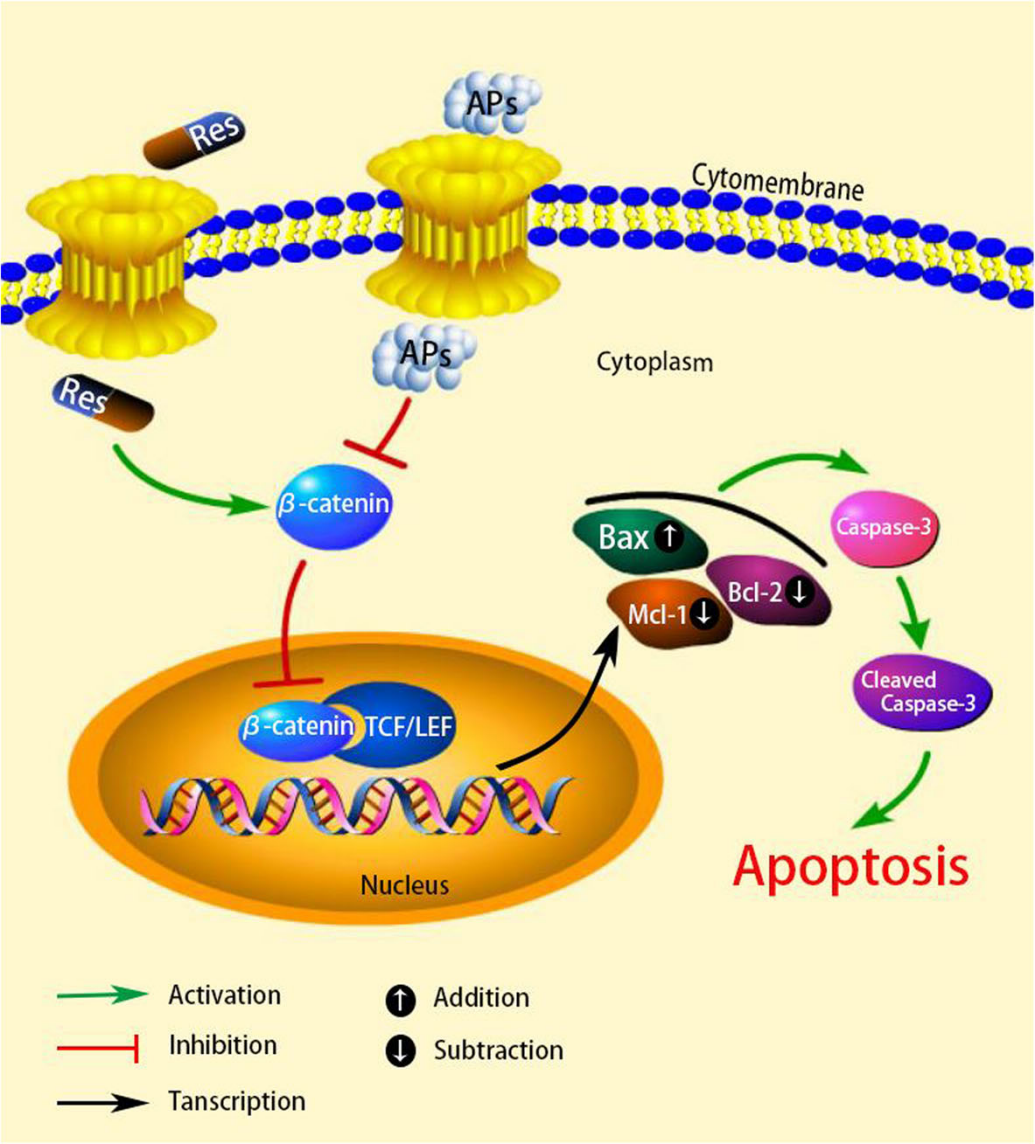
Schematic representation mechanism of APs inducing OB cells apoptosis. The Aps inhibited Wnt/β-catenin signal pathway, as a result, Bcl-2, Mcl-1 (antiapoptotic protein) transcript reduced and Bax, (proapoptotic protein) transcripted elevated. Then, Caspase-3 was activated. Eventually, this led to OB cells apoptosis. On the contrary, resevratrol could reverse the above effects

Our study suggested that APs may induce osteoblast apoptosis through decrease of antiapoptotic protein expression and increase of proapoptotic protein expression related to decreased β-catenin expression and restrained β-catenin translocated into the nucleus which a committed step in the wnt/β-catenin signal pathway, and resevratrol could prevent APs-induced osteoblast apoptosis through increased β-catenin expression (Fig. 5).

## Abbreviations

APs: Atypical antipsychotics
BCL-2: B cell lymphoma 2
BMD: Bone mineral density
CCK8: Cell counting kit-8
FCM: Flow cytometry
FITC: Annexin V-fluorescein isothiocyanate
LiCl: Lithium chloride
MOMP: Membrane permeabilization
OB: Osteoblast cell line hFob1. 19
PI: propidiμm iodide
TCF/LEF: T cell factor/ lymphocyte growth factor
WB: Western blot analysis
